# From environmental sensing to developmental control: cognitive evolution in dictyostelid social amoebas

**DOI:** 10.1098/rstb.2019.0756

**Published:** 2021-03-15

**Authors:** Pauline Schaap

**Affiliations:** School of Life Sciences, University of Dundee, Dundee DD15EH, UK

**Keywords:** excitable networks, self-organization, stress response, cAMP oscillations, *Dictyostelium*

## Abstract

Dictyostelid social amoebas respond to starvation by self-organizing into multicellular slugs that migrate towards light to construct spore-bearing structures. These behaviours depend on excitable networks that enable amoebas to produce propagating waves of the chemoattractant cAMP, and to respond by directional movement. cAMP additionally regulates cell differentiation throughout development, with differentiation and cell movement being coordinated by interaction of the stalk inducer c-di-GMP with the adenylate cyclase that generates cAMP oscillations. Evolutionary studies indicate how the manifold roles of cAMP in multicellular development evolved from a role as intermediate for starvation-induced encystation in the unicellular ancestor. A merger of this stress response with the chemotaxis excitable networks yielded the developmental complexity and cognitive capabilities of extant Dictyostelia.

This article is part of the theme issue ‘Basal cognition: conceptual tools and the view from the single cell’.

## 1. Introduction

A hallmark of all life is its ability to adapt continuously to a changing environment. This ability involves perceiving the change and performing an appropriate response. At the cellular level the response can be rapid and only require a change in protein function. The response can be somewhat slower, requiring a change in the available protein repertoire by gene expression, or it can occur over several generations by changing the genes themselves in the course of natural selection.

The process of perceiving environmental stimuli and mounting an appropriate response shares many properties with cognition in higher animals, such as (i) discrimination—the ability to recognize only those features that are relevant for species survival, (ii) memory—the ability to retain the perceived information for a persistent change in cell function, (iii) problem solving—the ability to select the most appropriate response, when faced with complex input, and (iv) communication—the ability to interact with members of the same or other species. In multicellular organisms these basal cognitive abilities evolved into more sophisticated behaviours that eventually incorporated anticipation, learning, sentience and creativity. The latter properties are generally only associated with organisms with a well-developed nervous system. However, to understand how they came into being, it is important to establish whether they are the result of entirely novel processes or whether they differ from environmental sensing only in a matter of degree.

While not claiming to answer that question, this review illustrates how mechanisms for finding food and responding to stress in a unicellular amoeba evolved into mechanisms controlling cell-type specialization and generation of form in its multicellular descendants, the Dictyostelia.

## 2. Multicellularity evolved many times

The well-known macroscopic multicellular organisms such as animals, plants and fungi start life as a single cell, which then enters into a series of cell divisions with all successive generations staying together to generate the adult form. Another form of multicellularity is more common and has evolved separately in seven out of the eight major divisions of eukaryotes [1]. In this form, a single cell emerges from a spore and starts feeding on smaller creatures or molecular substrates. The individual cells divide and stay apart as long as food is available. However, when starved they come together to construct a multicellular fruiting structure, where all or most of the cells differentiate into spores.

**Figure 1.**
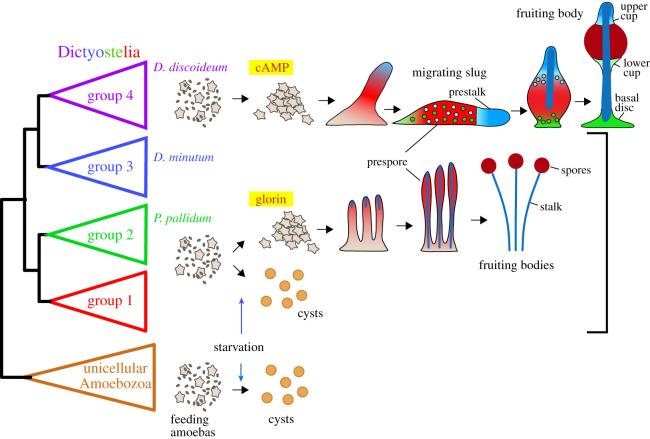
Life cycles of solitary and social amoebas. The eukaryote division Amoebozoa consists mainly of unicellular amoebas that enter into a dormant encapsulated cyst stage when experiencing starvation or other forms of stress. Instead, the dictyostelid social amoebas aggregate to form fruiting bodies with dormant encapsulated spores. Taxon groups 1, 2 and 3 mostly use glorin as attractant, and all cells in the aggregate first differentiate as prespore cells, with those at the tip re-differentiating into stalk cells. Many species in these groups can also still form cysts. Group 4 Dictyostelia lost encystation, but gained an intermediate migrating slug stage, and differentiation of prestalk cells and of three new cell types to support the stalk and spore head. This group uses cAMP as attractant for aggregation.

The dictyostelid social amoebas are, with over 150 known species, the largest group of such organisms. They are members of the Amoebozoa, a eukaryote division that otherwise almost exclusively consists of single-celled amoebas that individually differentiate into dormant cysts when starved. The Dictyostelia are subdivided into four major groups, which show different levels of morphological and behavioural complexity (figure 1). Species in groups 1, 2 and 3 differentiate into at most two cell types, stalk cells and spores, and form relatively small clustered or branched fruiting bodies. Many species in these groups have retained the ability to form solitary cysts under wet and dark conditions that do not favour aggregation [2].

The group 4 Dictyostelia form larger solitary fruiting bodies with up to three more somatic cell types. They show extensive light-oriented migration of an intermediate stage, the sorogen or slug. In its soil habitat, this serves to bring the cell mass to the top layer of the soil, where it then proceeds to form the fruiting body. Within the slugs, cells differentiate into prestalk and prespore cells and regulate the proportions of these cell types to the requirements for stalk cells and spores in the fruiting body (figure 1). This is in contrast to species in groups 1–3, where all cells differentiate into prespore cells, and then transdifferentiate into stalk cells at the top of the sorogen [3]. Group 4 species all use cyclic AMP (cAMP) as attractant for aggregation, whereas the dipeptide glorin and other attractants are used in groups 1–3. However, group 4 species have lost the ability to encyst.

## 3. Excitable networks enable chemotaxis and self-organization

The ability to detect and move towards food could be the most ancient sign of cognition and is in Dictyostelia and their amoeboid relatives achieved by chemotaxis—movement guided by the presence of chemical gradient. Chemotaxis is a complex process, since it requires the cell to detect a small concentration difference over a wide range of ambient concentrations. This is achieved by including adaptation in the response, which desensitizes the cell to the average concentration of attractant, while allowing response to change.

True to their name (amoibē = change) amoebas are always moving by extending pseudopods in different directions. These shape changes are the result of waves of polymerized actin (F-actin) that propagate through the cell cortex and cause pseudopod protrusion. The F-actin waves originate at sites where the cell touches the substratum. Such contacts locally activate the small GTPase Ras and increase levels of the membrane phospholipid PI(3,4,5)P3. Together with the 3-phosphatase PTEN, which dephosphorylates PI(3,4,5)P3, coronin, which depolymerizes F-actin and other factors, these components make up the cytoskeletal excitable network that governs wave initiation, propagation and extinction [4,5]. Using the same network, F-actin waves are also triggered when amoebas contact bacteria, but then lead to formation of cup-shaped protrusions that capture and internalize the bacteria.

**Figure 2.**
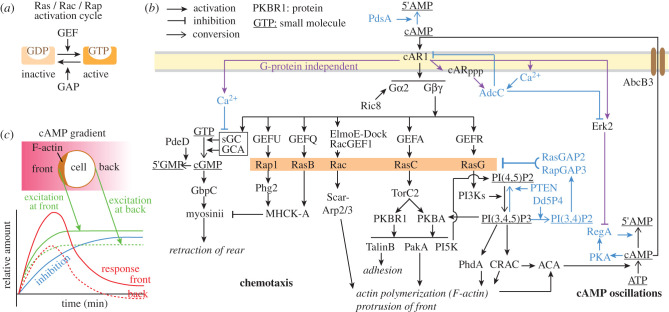
Excitable networks in chemotaxis and morphogenetic signalling. (*a*) Activation cycle of small GTPase molecular switches. GTP: guanosine triphosphate; GDP: guanosine diphosphate; GAP: GTPase activating protein; GEF: guanine nucleotide exchange factor. (*b*) Chemotactic signal processing. See main text for explanation. Protein functions/abbreviations: AdcC: arrestin C; ACA: adenylate cyclase A; CRAC: cytosolic regulator of ACA; cAR1: cAMP receptor; cARppp: C-terminally phosphorylated cAR1; Gα2, β, γ: heterotrimeric G-protein subunits; Ric8: GEF for Gα2; PdsA and RegA: cAMP phosphodiesterases; PdeD: cGMP phosphodiesterase; sGC and GCA: guanylate cyclases; GbpC: cGMP binding protein; Phg2, TorC2, MHCK-A, PKBA, PKBR1, PakA, ERK2 and PKA: protein kinases; PI3K and PI5K: phosphatidyl-inositol kinases; PTEN and Dd5P4: phosphatidyl-inositol phosphatases; ElmoE-Dock: complex with GEF function. Scar: adaptor linking the Arp2/3 actin nucleation complex to Rac; PhdA: actin regulating PH-domain protein. cAMP/cGMP: 3′5′-cyclic adenosine/guanosine monophosphate; 5′AMP/5′GMP: 5′-adenosine/guanosine monophosphate; AbcB3: ABC transporter B3. Violet arrows: cAR1 activated responses that do not require a heterotrimeric G-protein. Blue text and arrows: components of negative feedback loops. (*c*) Local activation and global inhibition govern actin polymerization. The chemotactic response is best explained by a Turing type reaction–diffusion model. Here stimulation with attractant causes rapidly spreading excitation and more slowly spreading inhibition. At the front of the cell, excitation exceeds inhibition and actin is polymerized, while at the back inhibition is dominant. Redrawn in modified form from [6].

In chemotaxis, the cytoskeletal excitable network interlinks with the signal transduction excitable network [5], which brings the localized polymerization of actin under control of the chemoattractant cAMP. cAMP activates the G-protein coupled receptor cAR1, which causes dissociation of the heterotrimeric GTPase G2 into its α and βγ subunits (figure 2). The βγ subunits activate at least six signalling cascades in parallel [7], which cause (i) actin polymerization resulting in pseudopod extension and adhesion to the substratum at the front of the cell, (ii) activation of myosinII at the back, which causes the cell to pull in its rear.

In most cascades this involves activation of small GTPases such as Rac, RasB, RasC, RasG and Rap1 [8]. Small GTPases are activated by guanine nucleotide exchange factors (GEFs), which assist dissociation of GDP from the inactive protein, allowing binding of GTP and activation. They are inactivated by GAPs (GTPase activating proteins), which activate their intrinsic GTPase activity causing GTP to convert into GDP (figure 2*a*).

Rac directly activates the actin nucleation complex Scar-Arp2/3 [9]. Many other small GTPases first activate protein kinases like Phg2 and TorC2, which, by phosphorylating other protein kinases like MHCK-A, PKBA, PKBR1 and ultimately PakA [10], regulate the activity of cytoskeletal components by phosphorylation. RasG activates phosphatidyl-inositol kinases (PI3Ks), which converts PI(4,5)P2 into PI(3,4,5)P3. PI(3,4,5)P3 acts as a membrane binding site for proteins with a PH domain, like PKBA, CRAC and PhdA [11], resulting in their recruitment from the cytosol to the plasma membrane, where they exert their function. CRAC has a dual role in actin polymerization and activation of adenylate cyclase A (ACA), the enzyme that synthesizes cAMP [12].

In addition to these responses which require the G-protein G2, binding of cAMP to cAR1 also triggers G-protein independent responses, such as activation of the protein kinase ERK2, transient influx of Ca^2+^, and phosphorylation of cAR1, which recruits the arrestin AdcC to the plasma membrane, where, activated by Ca^2+^, AdcC causes internalization and degradation of cAR1 as well as inhibition of ERK2 [13,14]. Ca^2+^ also inhibits the synthesis of cGMP, a small molecule required for myosinII activation [15].

ERK2 participates in a negative feedback loop, where cAMP, by successively activating PKA and the cAMP phosphodiesterase RegA, stimulates its own hydrolysis [16]. Together with the positive feedback loop formed by cAMP stimulating its own synthesis by cAR1, RasC, PI3 K and CRAC mediated activation of ACA [12], and PdsA mediated cAMP hydrolysis outside the cell, these interactions represent a separate excitable network, which is proposed to generate the spontaneous cAMP oscillations that control the supra-cellular processes of aggregation and morphogenesis.

The interactions between the components that control localized actin polymerization are highly dynamic. Generally speaking, the components that act positively on actin polymerization, such as TorC2, PI3K, PI(3,4,5)P3, PKBA/PKBR1, PhdA and CRAC, self-organize to take up position at the side of the cell that faces the highest cAMP concentration, while negatively acting components such as PTEN, Dd5P4 and several GAPs take up positions at the back of the cell [5,17]. The first set is interconnected in a fast positive feedback loop acting on the activation state of small GTPases like Ras and Rap, while the second set is part of a slower negative feedback loop that inhibits Ras and Rap. When a naive cell is exposed to a cAMP gradient, rapid excitation of actin polymerization is triggered throughout the cell, followed by slower inhibition (figure 2*c*). At the back of the cell, where the perceived cAMP concentration is lower, both the excitation and inhibition response are lower, causing excitation at the back to be overwhelmed by inhibition emanating from the front. Only at the front, excitation exceeds inhibition sufficiently to sustain persistent actin polymerization and pseudopod extension [7].

There are more negative feedback loops in the cAMP signal transduction cascade, such as inhibition of cAMP-induced cGMP synthesis by cAMP-induced Ca^2+^-influx [15], cAMP-induced internalization of cAR1 in response to cAMP-induced cAR1 phosphorylation, and activation of arrestin [14]. Additionally, there is considerable redundancy in the pathways leading to actin polymerization. The significance of either is at present not fully understood. It should be realized that in their natural environment cells do not perceive just one signal at a time, as outlined here, but are simultaneously beset by a panoply of sensory input. Since their continued survival depends on appropriate distinction between and responses to different stimuli, the biochemical computational skills of a single amoeba have to be quite considerable.

## 4. cAMP oscillations self-organize aggregation, morphogenesis and slug movement

Some small species of Dictyostelia, such as *Dictyostelium minutum* or *Dictyostelium lacteum* in group 3 secrete chemoattractant continuously causing surrounding cells to move individually towards the source. The cAMP oscillatory network, as used by *Dictyostelium discoideum*, is a more effective mechanism for aggregation. Firstly, an initial cAMP pulse causes surrounding cells to synthesize a cAMP pulse and secrete it using the ABC transporter AbcB3 [18], thus propagating an unattenuated wave of cAMP through the population [19]. Owing to cAMP hydrolysis by PdsA, each pulse presents the cell with a steep gradient, instead of the shallow gradients that result from secretion by a single source.

Once aggregated, the tip of the aggregate continues to emit cAMP pulses [20]. The propagating cAMP waves cause the cells to move upward, aerially projecting the structure as the sorogen or ‘slug’. At this stage the slug can either directly form the fruiting body or fall over and start migrating. In nature, slugs move towards the soil surface by responding to light and warmth. While the slug does not possess sensory organs, it does this as follows. The rounded slug front acts as a lens focusing infrared and visible light on the distal surface. This increases metabolism at this spot, resulting in enhanced NH_3_ production by protein degradation [21]. NH_3_ inhibits cAMP signalling [22], causing the oscillator to move towards the light-facing side of the slug and thereby turning slug movement towards the light. After having reached the soil surface, the slug projects the tip upward in response to incident light and starts to form the fruiting body.

By organizing the motility of individual cells to generate form and movement of the multicellular structure, the cAMP excitable network functions analogously to the animal nervous system, where the propagation of an action potential along an axon represents another excitable network, which in this case activates muscle fibre contraction and movement of the organism. One reason why the cAMP excitable network is not more commonly used is that by depending on cAMP diffusion, at 5 × 10^−6^ cm^2^ s^−1^, it is orders of magnitude slower than an action potential moving along a motor nerve at approximately 40 m s^−1^. Nevertheless, the cAMP excitable network imparts a range of adaptive behaviours to what is essentially a heap of amoebas, highlighting the power of nonlinear biochemical networks to generate apparent cognitive function.

## 5. Extensive use of cAMP signalling in the *Dictyostelium* differentiation programme

**Figure 3.**
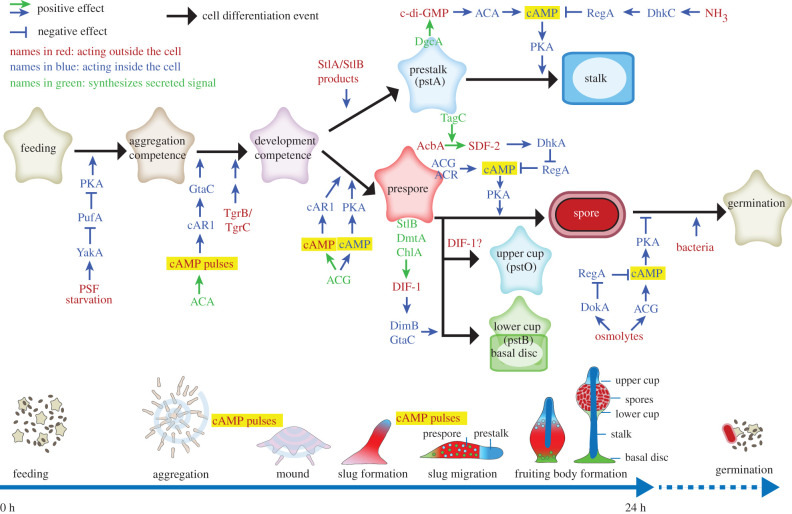
Signals that regulate the *D. discoideum* life cycle. Multicellular development is initiated by starvation and accumulation of the quorum sensing factor PSF, which act to upregulate the translation of cAMP dependent protein kinase (PKA). The amoebas aggregate by secreting cAMP pulses to form multicellular mounds. The mound tip continues to emit cAMP pulses and attracts cells from underneath, causing slugs and fruiting bodies to form. In the slug, cAMP acting on both cAMP receptors (cAR1) and PKA induces prespore differentiation, while prespore cells synthesize signals like DIF-1, which cause cells to differentiate into stalk, basal disc and upper and lower cup precursors. Loss of NH_3_ from the slug tip prevents cAMP hydrolysis by the cAMP phosphodiesterase RegA, thus facilitating induction of stalk formation by c-di-GMP activation of ACA and PKA. Prespore cells secrete the protein AcbA, which is cleaved by TagC on prestalk cells, to produce SDF-2, which activates PKA by inhibiting RegA. High solute levels in the spore head prevent spore germination by activating the osmosensors ACG and DokA, which synthesize cAMP and inhibit RegA, respectively, thereby also activating PKA. ACA: adenylate cyclase A; ACG: adenylate cyclase G; ACR: adenylate cyclase R; cAMP: 3′5′-adenosine monophosphate; c-di-GMP: 3′,5′-cyclic diguanylic acid; ChlA: halogenase chlorination A; DgcA: diguanylate cyclase A; DhkA: histidine phosphatase A; DhkC: histidine kinase C; DIF-1: differentiation inducing factor 1; DimB: transcription factor DIF-insensitive mutant B; DmtA: des-methyl-DIF-1 methyltransferase; DokA: osmosensing histidine phosphatase; GtaC: GATA-binding transcription factor C; NH_3_: ammonia; PufA: Pumilio RNA binding protein; SDF-2: spore differentiation factor 2; StlA: polyketide synthase Steely A; StlB: polyketide synthase Steely B; Tgr: transmembrane, IPT, IG, E-set, repeat protein; YakA: DYRK family protein kinase.

cAMP also plays a dominant role in the spatio-temporal control of cell differentiation during the developmental programme (figure 3). As part of a gene-regulatory positive feedback loop on the aggregation process, the cAMP pulses that mediate aggregation also upregulate expression of the genes that are required for aggregation, such as *cAR1*, *ACA* and *pdsA* [23], and render cells competent for subsequent induction of post-aggregative genes [24]. Owing to close cell proximity, the extracellular cAMP concentration increases once cells are aggregated, with micromolar cAMP now triggering prespore differentiation [25]. Prespore cells lose chemotactic responsiveness [26] and secrete factors like DIF-1, which prevent neighbouring cells from acquiring a prespore cell fate [27]. These so-called anterior-like cells (ALCs) chemotax preferentially towards the tip to form the prestalk region, which makes up the anterior 25% of the slug. Here they sustain a population capable of generating cAMP pulses [28], with the more distal ALCs propagating the signal.

In addition to its roles as secreted signal, cAMP has many intracellular roles as second messenger for other external stimuli, where it activates cAMP dependent protein kinase (PKA). PKA is required for the transition from growth to development by triggering basal expression of aggregation genes [29]. Together with cAMP activation of cAR1, cAMP activation of PKA induces expression of prespore genes [30]. PKA activation is also required for the maturation of spore and stalk cells [31] and to prevent spores from germinating in the fruiting body [32].

Both stalk cells and spores become encased in rigid cell walls as part of their differentiation process. Fruiting body formation depends on amoeboid movement, which is obviously impaired by rigid cell walls. It is therefore important that spores and stalk cells mature only at the right time and place. Spatial control over stalk formation is achieved by interaction between secreted c-di-GMP and ACA [33]. c-di-GMP is synthesized by a prokaryote-type diguanylate cyclase that is expressed by all prestalk cells [34], while ACA is preferentially expressed at the utmost tip [28]. c-di-GMP is a potent activator of ACA, with cAMP then activating PKA to induce stalk maturation. Owing to the localized expression of ACA, this only occurs at the slug tip, where prestalk cells normally initiate stalk formation, thereby synchronizing organization of cell movement with organization of cell differentiation.

The timely maturation of spores is achieved by intensive communication between prespore cells and the maturing stalk. Here the intracellular cAMP phosphodiesterase RegA plays a central role [35]. Apart from a cAMP-phosphodiesterase domain, RegA contains a response regulator domain that requires phosphorylation of a conserved aspartate for the phosphodiesterase to be active [36]. RegA phosphorylation/dephosphorylation occurs by the conserved histidine–aspartate phosphorelay system which is activated by sensor histidine kinases/phosphatases (SHKPs) [37]. *Dictyostelium* has 16 SHKPs, of which many have known biological roles, but I will here only highlight the roles of two of those, DhkC and DhkA.

DhkC is a sensor for NH_3_ [38], which is produced by autophagy in the starving cells. The migrating slug is partially submerged in its own NH_3_, but the aerial projection of the slug tip in response to incident light enables dissipation of NH_3_ gas. NH_3_ prevents stalk maturation by activating DhkC, which, by phosphorylating and thereby activating RegA, inhibits cAMP accumulation and activation of PKA. Loss of NH_3_ from the projecting tip relieves this inhibition, allowing stalk maturation to proceed.

In the case of DhkA, it is the phosphatase and not the kinase activity that is activated by its ligand SDF-2 (spore differentiation factor 2) [39]. The peptide SDF-2 is produced through cleavage of acyl-CoA binding protein (AcbA), which is secreted by prespore cells [40], by the protease TagC, which is exteriorly exposed on prestalk cells. By activating DhkA on prespore cells, SDF-2 causes reverse phosphorelay from RegA, rendering its cAMP phosphodiesterase inactive and allowing PKA and thereby spore maturation to be activated.

cAMP is extensively used as a second messenger by mammals to regulate many aspects of metabolism, physiology, development and memory and also regulates diverse cellular functions in all other domains of life. However, its role is never as dominant as it appears to be in *D. discoideum,* where its entwined regulation of cell motility and cell differentiation controls the entire developmental programme.

## 6. An ancestral role for cAMP in amoebozoan stress sensing

Comparative genomics, combined with gene knock-out and gene replacement, provide opportunities for understanding how cAMP signalling networks evolved. The genes encoding PKA, ACG, ACR and RegA, which in *D. discoideum* detect, synthesize and hydrolyse intracellular cAMP, are conserved across the four dictyostelid taxon groups (figure 1) and, apart from ACG, also in the unicellular Amoebozoa *Protostelium fungivorum*, *Physarum polycephalum* and *Acanthamoeba castellani* [41]. Similar to *D. discoideum*, PKA activation is required for aggregation and fruiting body formation in *Polysphondylium pallidum* in taxon group 2, and, remarkably, also for encystation of single amoebas [42,43]. As is the case for sporulation, deletion of *PKA* or of *ACR* and *ACG* together prevents encystation, while deletion of *regA* causes precocious encystation, while amoebas are still feeding [43,44]. Also in the distantly related amoebozoan, *A. castellani,* RegA inhibition causes cells to encyst while food is still plentiful.

This indicates that the roles of PKA, ACR, ACG and RegA in spore and stalk maturation are evolutionarily derived from a role in encystation, where starvation or drought acts to elevate intracellular cAMP [45], which then acts on PKA to induce encystment. While involvement of a specific sensor histidine kinase/phosphatase in encystation is not yet known, the genomes of the solitary Amoebozoa listed above each contain a large repertoire of these enzymes. Regulation of RegA activity by phosphorelay is therefore very possible.

## 7. Extracellular cAMP signalling is unique to Dictyostelia

The genes involved in the extracellular roles of cAMP, such as *cAR1* and *pdsA*, are conserved throughout Dictyostelia, but are not present in the solitary Amoebozoa. *Dictyostelium discoideum* has four *cAR* genes, but of those only *cAR1*, which mediates chemotaxis and prespore gene induction by cAMP, is conserved outside group 4 [46]. *Polysphondylium pallidum* has two *cAR1*-like genes and deletion of both does not affect aggregation [47], for which *P. pallidum* uses glorin as attractant. However, without cARs, fruiting body morphogenesis is stunted, with disorganized differentiation of stalk cells. Remarkably, the remaining cells differentiate into cysts instead of spores. Without cARs, cAMP cannot induce prespore gene expression, but because PKA is still activated, the starving cells form cysts instead. This again illustrates that sporulation is evolutionarily derived from encystation [47].

Deletion of the extracellular phosphodiesterase *pdsA* in *P. pallidum* disrupted fruiting body morphogenesis similarly to deletion of *cAR*s [48]. Since PdsA specifically sustains oscillatory cAMP signalling by hydrolysing cAMP between pulses, this indicates that cell movement during *P. pallidum* post-aggregative development is organized by cAMP oscillations, as was found earlier for the group 3 species *D. minutum* [49]. This indicates that the cAMP oscillatory network was first used to organize fruiting body morphogenesis, before group 4 used it for aggregation as well. The organization of the promoters of the *cAR1*, *pdsA* and *ACA* genes indicates how this happened. All three genes have multiple promoters; the promoter closest to the coding sequence directs transcription during post-aggregative development, while a more distal promoter directs expression during aggregation [50–52]. At least for *cAR1*, this distal promoter is absent outside group 4 [46]. Apparently, addition of distal aggregative promoters to existing cAMP signalling genes enabled group 4 to use cAMP as attractant for aggregation.

cAR1 function itself did not change, since a group 3 cAR1 restores cAMP oscillations in a *D. discoideum* cAR null mutant [46]. This is not the case for PdsA, which increased its affinity for cAMP 200-fold in group 4, likely to accommodate the lower cAMP concentrations in a dispersed field of amoebas. *Dictyostelium discoideum* secretes a PdsA inhibitor, PDI, during aggregation, which generates discontinuities that favour the formation of spiral cAMP waves over concentric waves, with the former being able to control larger aggregation territories [53]. PDI belongs to a family of extracellular matrix proteins that are present in all Dictyostelia. However, in its current modified form, it is only present in group 4 [48], suggesting that co-option of this matrix protein as a PdsA inhibitor may at least partially be responsible for the typically large and robust fruiting bodies in this group [2].

## 8. *Dictyostelium* development—a merger of the chemotactic network with a stress response?

Across the approximately nine divisions of eukaryotes, the amoeboid mode of cell movement is most common and likely to be ancestral to all eukaryotes. Many components of the cytoskeletal and signal transduction excitable networks that trigger and direct actin waves, respectively, are conserved between *D. discoideum* and mammalian cells [5]. While in amoebas the actin waves are involved in the formation of pseudopods and phagocytic cups, in mammalian cells they generate a range of protrusions, such as lamellipodia, filopodia, invadopodia and podosomes. In view of the fact that Dictyostelia and mammals evolved multicellularity independently from each other, this implies that the cytoskeletal and signal transduction excitable networks have ancient origins that pre-date the evolution of multicellularity.

The differentiation pathway of encystation is equally ancient and prevalent among the unicellular members of all eukaryote divisions [54]. Encapsulation as a dormant cyst is the preferred and often only strategy available to single cells to survive environmental stress, such as starvation, drought or, for phytoplankton, lack of light in winter at high latitudes. In Dictyostelia, starvation and drought stress trigger encystation by increasing intracellular cAMP and activating PKA [43,45,54], with the cAMP phosphodiesterase RegA maintaining low cAMP levels in cells that are not under stress [44]. Evidence for RegA and PKA involvement in other Amoebozoa is more sporadic [44,55,56], which is largely due to lack of gene modification procedures for these organisms.

While unicellular Amoebozoa lack cAMP receptors and are therefore unlikely to use cAMP as chemoattractant, in *D. discoideum*, the cAMP pathway has become entangled with the signal transduction excitable network that controls the chemotactic response (figure 2). Specifically, the regulation of the ACA by RasG mediated PI(3,4,5)P3 production, combined with regulation of cAMP levels by PKA activation of RegA, has uniquely generated the cAMP excitable network in Dictyostelia that coordinates the supra-cellular processes of aggregation and morphogenesis.

**Figure 4.**
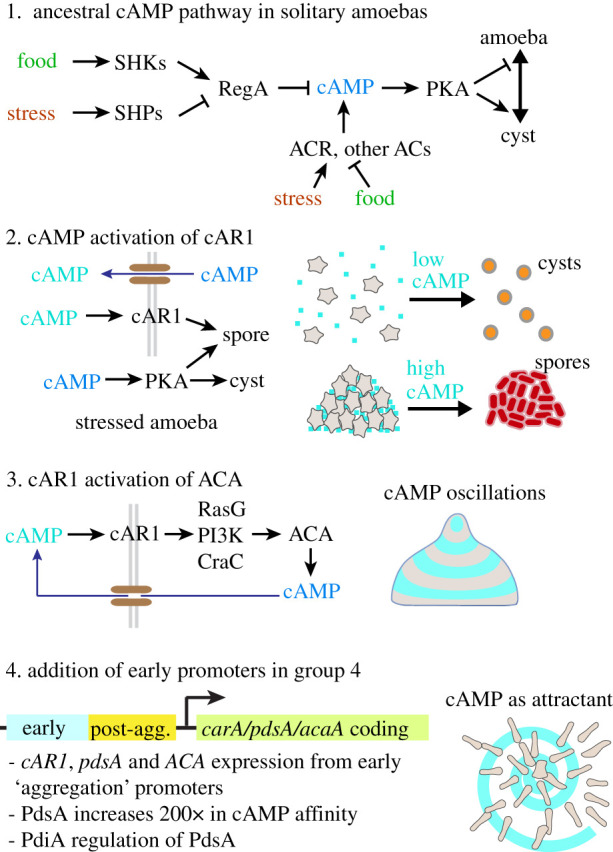
Evolution of cAMP signalling in Dictyostelia. Possible scenario for the evolution of cAMP signalling in *D. discoideum* from an amoebozoan stress response. See main text for explanation.

The comparative evolutionary studies described in the previous paragraph suggest how this may have happened (figure 4):

1. An ancestral pathway was present in solitary Amoebozoa, where cAMP, PKA and RegA acted as intermediates for sensing stress or for conditions favourable for growth by sensor histidine kinases/phosphatases and/or sensor-linked adenylate cyclases. In Dictyostelia these sensors evolved to detect developmental signals that thereby acted on PKA to control the maturation of spores and stalk cells in the fruiting body.
2. Early colonial dictyostelids used accumulation of secreted cAMP as a sensor for the aggregated state, causing cells to differentiate into spores when detecting elevated levels of both intra- and extracellular cAMP, and into cysts when only detecting a rise in intracellular cAMP.
3. Next, cAMP receptors, ACA, RegA and PKA became incorporated in the chemotaxis excitable network, enabling cells to generate the cAMP oscillations that coordinate the morphogenesis of well-structured fruiting bodies.
4. Finally, the cAMP oscillatory genes *cAR1*, *pdsA* and *ACA* became expressed before and during aggregation by the addition of distal ‘early’ promoters to the existing ‘post-aggregative’ promoters Other modifications, such as increased affinity of PdsA for cAMP and secretion of the PdsA inhibitor PDI imparted greater efficiency to cAMP signalling in a dispersed field of amoebas.

This scenario is speculative and contains many missing links. However, it helps to perceive how a mundane stimulus–response pathway can evolve to incorporate interactions that impart positive and negative feedback and thereby turn it into an excitable network with flexible regulatory potential. In Dictyostelia, the merger of at least two of these networks enabled a collection of stressed amoebas to self-organize into a motile multicellular structure, capable of stimulus-driven decision-making.

## Data accessibility

This article has no additional data.
